# X-ray Reciprocal Space Mapping of Graded Al_*x*_Ga_1 − *x*_N Films and Nanowires

**DOI:** 10.1186/s11671-016-1299-7

**Published:** 2016-02-09

**Authors:** Hryhorii V. Stanchu, Andrian V. Kuchuk, Vasyl P. Kladko, Morgan E. Ware, Yuriy I. Mazur, Zbigniew R. Zytkiewicz, Alexander E. Belyaev, Gregory J. Salamo

**Affiliations:** V. Lashkaryov Institute of Semiconductor Physics, National Academy of Sciences of Ukraine, Pr. Nauky 41, 03680 Kyiv, Ukraine; Institute for Nanoscience and Engineering, University of Arkansas, West Dickson 731, Fayetteville, AR 72701 USA; Institute of Physics, Polish Academy of Sciences, Al. Lotnikow 32/46, 02-668 Warsaw, Poland

**Keywords:** Al_*x*_Ga_1 − *x*_N, Graded films and nanowires, Kinematical theory, Asymmetrical RSM

## Abstract

The depth distribution of strain and composition in graded Al_*x*_Ga_1 − *x*_N films and nanowires (NWs) are studied theoretically using the kinematical theory of X-ray diffraction. By calculating $\left(20\overline{2}5\right)$ reciprocal space maps (RSMs), we demonstrate significant differences in the intensity distributions from graded Al_*x*_Ga_1 − *x*_N films and NWs. We attribute these differences to relaxation of the substrate-induced strain on the NWs free side walls. Finally, we demonstrate that the developed X-ray reciprocal space map model allows for reliable depth profiles of strain and Al composition determination in both Al_*x*_Ga_1 − *x*_N films and NWs.

## Background

The demonstration of p-type doping through the so-called polarization doping technique for Al_*x*_Ga_1 − *x*_N alloys is finding more and more practical applications in modern optoelectronic devices. Using compositionally graded Al_*x*_Ga_1 − *x*_N films, polarization-induced p-n junctions and light emitting diodes (LEDs) have been successfully fabricated [1–3]. This fundamentally new type of p-n junction allowing deep ultraviolet LEDs was shown for graded Al_*x*_Ga_1 − *x*_N catalyst-free nanowires (NWs) without the use of impurity doping [4, 5]. Such doping enhancement occurs from grading the composition of Al_*x*_Ga_1 − *x*_N alloys along the *c*-axis and thus grading the magnitude of the intrinsic polarization in the wurtzite crystal structure. This effectively forms an uncompensated space charge field which is neutralized by free charges of the opposite sign. These free charges become the free electrons and holes which make up a device structure.

The depth profile of the aluminum content is therefore the key factor in controlling the properties of graded Al_*x*_Ga_1 − *x*_N alloys. However, even for the same Al depth profiles, there is a significant difference between the properties of graded planar films and NWs. The different in- and out-of-plane strain profiles resulting from the free surface of the NWs lead to a difference in (i) the polarization-induced doping carrier densities through differences in the piezoelectric component of the polarization and (ii) the strain-related defect density. Moreover, the large ratio of surface to bulk states can enhance the polarization doping efficiency for graded NWs. In addition, it should be noted that there is also a difference between the strain profiles of catalyst-free and top-down fabricated NWs obtained by dry etching if dislocations form as a result of plastic strain relaxation in the pre-etched material. Therefore, tuning the properties of graded Al_*x*_Ga_1 − *x*_N films and NWs requires rapid and reliable techniques to determine chemical composition and strain depth profiles.

High resolution X-ray diffraction (HRXRD) is a nondestructive technique that permits rapid determination of the chemical composition, strain state, and thickness of epitaxial films. An X-ray method for measuring the composition and relaxation depth profiles in In_*x*_Ga_1 − *x*_As/GaAs and GaAs_1 − *x*_P_*x*_/GaAs graded epitaxial films has been demonstrated in [6]. In [7], the kinematical theory of X-ray diffraction is applied for strain and composition depth profile determination in pseudomorphically grown graded Al_*x*_Ga_1 − *x*_N/GaN epitaxial films by the simulation of the ω/2θ X-ray diffraction profiles. In this study, we extend the HRXRD method to the case of graded Al_*x*_Ga_1 − *x*_N NWs through the calculation of asymmetrical reciprocal space maps (RSMs). Particular attention is paid to the in-plane lattice parameter relaxation in Al_*x*_Ga_1 − *x*_N NWs due to the free sidewalls and how this compares with continuous coherently grown Al_*x*_Ga_1 − *x*_N films.

## Methods

For compositionally graded Al_*x*_Ga_1 − *x*_N films and NWs, both strain and Al distributions affect the alloy’s lattice parameters and complicate the determination of composition and strain profiles. Moreover, the strain relaxation process in graded Al_*x*_Ga_1 − *x*_N films differs from that in NWs, which requires a different approach.

In the case of graded Al_*x*_Ga_1 − *x*_N films, an approach for the determination of the depth profiles of the biaxial strain and the Al composition was proposed in [6]. It requires the measurement of at least two X-ray reflections to give the in-plane lattice parameter (*a*_*F*_), which allows the out-of-plane, *ε*_⊥_(*t*), and in-plane, *ε*_∥_(*t*), strain distribution for an Al composition profile of coherently grown graded Al_*x*_Ga_1 − *x*_N film (F) to be determined:

1 $$ {\varepsilon}_{\perp }(t)=\frac{c_F(t)-{c}_0(t)}{c_0(t)} $$

2 $$ {\varepsilon}_{\parallel }(t)=\frac{a_F-{a}_0(t)}{a_0(t)} $$

where *c*_*F*_(*t*) is the strained, out-of-plane lattice parameter and *c*_0_(*t*) and *a*_0_(*t*) are respectively the fully relaxed out-of-plane and in-plane lattice parameters of the Al_*x*_Ga_1 − *x*_N film at depth *t*.

For the case of biaxial strain, the ratio between the *ε*_⊥_(*t*) and *ε*_∥_(*t*) for wurtzite crystals is given by

3 $$ {\varepsilon}_{\perp }(t) = - 2{\varepsilon}_{\parallel }(t)\frac{C_{13}(t)}{C_{33}(t)} $$

where *C*_13_(*t*) and *C*_33_(*t*) are the elastic constants of the fully relaxed Al_*x*_Ga_1 − *x*_N film at depth *t*. The relaxed lattice parameters and the elastic constants of the graded Al_*x*_Ga_1 − *x*_N structure are assumed to vary linearly with the composition, *x*_Al_ (Vegard’s law).

The substitution of Eqs. (1) and (2) in (3) results in the depth profile of the lattice parameter *c*_*F*_(*t*) of a coherently grown graded Al_*x*_Ga_1 − *x*_N film:

4 $$ {c}_F(t)={c}_0(t)\left(1-2\frac{C_{13}(t)}{C_{33}(t)}\frac{a_F-{a}_0(t)}{a_0(t)}\right) $$

**Fig. 1:**
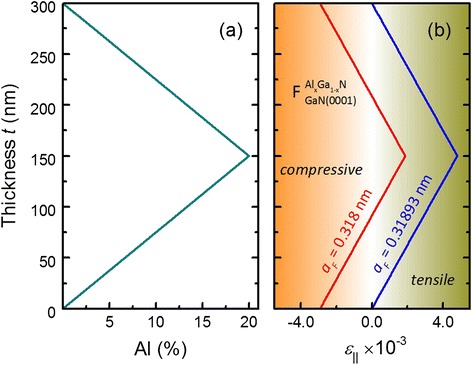
Depth profiles of the Al composition (**a**) and the in-plane strain (**b**) of graded Al_*x*_Ga_1 − *x*_N films. In **b**, the *red* and *blue curves* indicate the *ε*_∥_(*t*) profiles of partially relaxed (*a*_*S*_ ≠ *a*_*F*_ = 0.318 nm) and fully strained graded Al_*x*_Ga_1 − *x*_N films (*a*_*S*_ = *a*_*F*_ = 0.31893 nm), respectively

Altogether, according to Eq. (4), after measurements of the in-plane lattice parameter (*a*_*F*_ = const) of coherently grown graded Al_*x*_Ga_1 − *x*_N film, the depth distribution of the biaxial strain can be evaluated given an Al composition depth profile. And, as mentioned above, the fitting of an experimental symmetrical or asymmetrical RSM will allow for the separation of the strain and Al composition profiles. This approach is valid both, for coherent films fully strained to the substrate (*a*_*F*_ = *a*_*S*_, where *a*_*S*_ is the substrate in-plane lattice parameter), i.e., pseudomorphic growth, and also for the case of partially relaxed films (*a*_*F*_ ≠ *a*_*S*_). It should be noted, that depending on the depth profile of the Al composition and the relaxation degree of the graded Al_*x*_Ga_1 − *x*_N film, the coherent growth on a GaN substrate results in a tensile and/or compressive strain, as demonstrated in Fig. 1.

The depth profile of the Al composition for 300 nm thick graded Al_*x*_Ga_1 − *x*_N film linearly increasing from *x*_Al_ = 0 % to *x*_Al_ = 20 % at the center of the film then linearly decreasing back to *x*_Al_ = 0 % is shown in Fig. 1a. For this Al distribution the in-plane strain, *ε*_||_(*t*), profiles are shown in Fig. 1b, calculated according to the Eq. (2) for fully strained and partially relaxed films. Here, we see that pseudomorphic growth of graded Al_*x*_Ga_1 − *x*_N on [0001] oriented GaN results in tensile in-plane strain along the entire film thickness. In contrast, the relaxation of a coherent graded Al_*x*_Ga_1 − *x*_N film can result in both tensile and compressive in-plane strains depending on the film’s relaxation degree and the Al composition profile.

It is well understood that misfit strain energy buildup during growth of the heteroepitaxial films and plastic relaxation via misfit dislocation generation can occur if the film thickness exceeds the Matthews–Blakeslee critical thickness [8]. In contrast, due to the free side walls of NW structures, the possibility of elastic strain relaxation exists allowing for a much larger misfit to build up with the substrate. However, the varying Al composition in graded Al_*x*_Ga_1 − *x*_N NWs will result in a large internal strain even if separated from the substrate, i.e., “free” NWs. To calculate this internal strain, we apply the approach described in [9] where the elastic strain of GaN and AlN layers of an GaN/AlN superlattice is calculated considering the minimum elastic energy of one period of the superlattice. Accordingly, we divide the entire Al_*x*_Ga_1 − *x*_N NW into sublayers with thickness (*l*). Then, the in-plane lattice parameter which minimizes the elastic energy is approximated as:

5 $$ \overline{a}=\frac{{\displaystyle {\sum}_t}{a}_0(t)K(t)}{{\displaystyle {\sum}_t}K(t)}, $$

where *K*(*t*) is given by

6 $$ K(t)=\frac{l}{a_l^2(t)}\left({C}_{11}(t)+{C}_{12}(t)-2\frac{C_{13}^2(t)}{C_{33}(t)}\right), $$

with the constants as before for the fully relaxed Al_*x*_Ga_1 − *x*_N sublayer at depth *t*. Therefore, the elastic in-plane strain in a graded Al_*x*_Ga_1 − *x*_N “free” NW is given by

7 $$ {\varepsilon}_{\parallel}^{\mathrm{free}}(t)=\frac{\overline{a}-{a}_0(t)}{a_0(t)}. $$

**Fig. 2:**
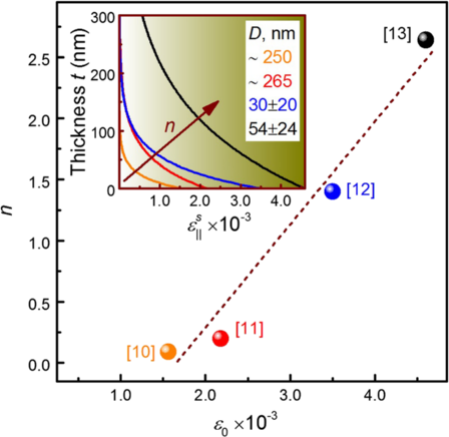
The dimensionless parameter *n* as a function of the initial strain *ε*_0_ at the NW/substrate interface taken from [12, 13] and extracted from data in [10, 11], with the *inset* representing the substrate induced in-plane strain profiles for GaN NWs on Si(111) substrate

An exponential decay of the substrate induced strain (${\varepsilon}_{\left|\right|}^S$) for GaN NWs on a Si substrate was experimentally demonstrated in [10–13]. In terms of the in-plane strain throughout the NWs, this is given as:

8 $$ {\varepsilon}_{\parallel}^S(t)={\varepsilon}_0{e}^{-\frac{t}{L}}, $$

where *ε*_0_ = (*a*_*s*_ − *ā*)/*ā* is the initial strain at the NW/substrate interface; *L* = *nD* is a characteristic relaxation depth for the NW; *D* is the diameter of the NW; and *n* is a dimensionless quantity which can be derived through the solution of the elastic problem for simple geometries [13], but for more complicated geometries, is used as a fitting parameter. In Fig. 2, we demonstrate the relationship between *ε*_0_ and the parameter *n* with data from strain analysis of GaN/Si(111) NWs formed by “top-down,” dry etching (~250 nm in diameter) [10, 11] as well as formed by catalyst-free “bottom-up” growth (~30–55 nm in diameter) [12, 13]. Here, the small *ε*_0_ and the large diameters of the top-down fabricated NWs compared with catalyst-free NWs indicate that some degree of plastic relaxation has occurred during the growth of the thick heteroepitaxial GaN layer. Thus, we can conclude that both the structural perfection as well as the diameter of a NW has a strong influence on the parameter *n*, which leads to different strain relaxation depths in GaN NWs fabricated with different techniques (see inset in Fig. 2). Since the relaxation depth determination is beyond the scope of this work and a more detailed experimental analysis of *n*(*ε*_0_) is not available, we will assume *n* = 1 for all subsequent calculations of ${\varepsilon}_{\left|\right|}^S(t)$.

Thus, considering ${\varepsilon}_{\left|\right|}^{\mathrm{free}}(t)$ and ${\varepsilon}_{\left|\right|}^S(t)$, the total in-plane strain in graded Al_*x*_Ga_1 − *x*_N NWs is given by

9 $$ {\varepsilon}_{\parallel}^{\mathrm{total}}(t)={\varepsilon}_{\parallel}^{\mathrm{free}}(t)+{\varepsilon}_{\parallel}^S(t)=\frac{a_{\mathrm{NW}}(t) - {a}_0(t)}{a_0(t)}, $$

which results in the depth profile of the in-plane lattice parameter, *a*_NW_(*t*) given by

10 $$ {a}_{\mathrm{NW}}(t)={a}_0(t)\left(1+{\varepsilon}_{\parallel}^{\mathrm{total}}(t)\right). $$

The depth profiles of the out-of-plane lattice parameter, *c*_NW_(*t*), for graded Al_*x*_Ga_1 − *x*_N NWs, can be calculated by substitution *a*_*F*_ = *a*_NW_(*t*) in Eq. (4).

**Fig. 3:**
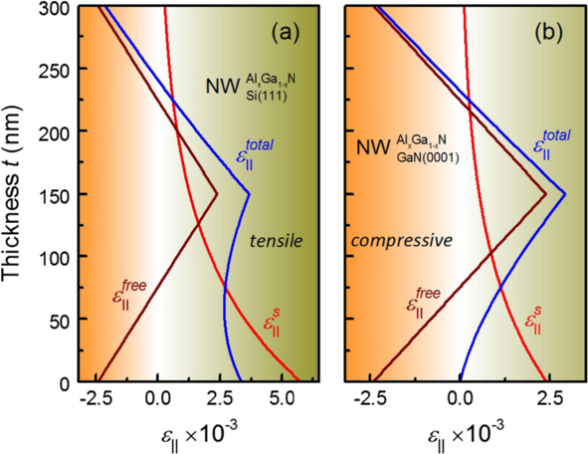
The ${\varepsilon}_{\left|\right|}^S(t)$ (*red solid curve*) and ${\varepsilon}_{\left|\right|}^{\mathrm{total}}(t)$ (*blue solid curve*) profiles of graded Al_*x*_Ga_1 − *x*_N NWs on **a** Si(111) and **b** GaN(0001) substrates with the *brown* *solid curves* indicating the ${\varepsilon}_{\left|\right|}^{\mathrm{free}}(t)$ profile. The Al composition profile along the NW length is the same as shown in Fig. 1a for the planar film

The depth profiles of the in-plane strain for 300-nm thick graded Al_*x*_Ga_1 − *x*_N NWs (100 nm in diameter) on Si(111) and GaN(0001) substrates are calculated according to Eq. (9) and presented in Fig. 3. The Al composition profile along the NW length is the same as shown in Fig. 1a for the planar film. In comparison with coherent films, the total strain ${\varepsilon}_{\left|\right|}^{\mathrm{total}}(t)$ in graded Al_*x*_Ga_1 − *x*_N NWs is dominated mostly by the substrate at the NW’s bottom, whereas the ${\varepsilon}_{\left|\right|}^{\mathrm{total}}(t)$ at the NW’s top is affected mostly by the Al composition. In general, by comparison of Fig. 1 and Fig. 3, there are strong differences between the strain profiles of graded Al_*x*_Ga_1 − *x*_N grown as thin films or grown as NWs with the same Al composition profiles. This should result in different reciprocal space X-ray intensity distribution for these objects.

The numerical calculation of RSMs is performed using the kinematical theory of X-ray diffraction, within the limits of which the reciprocal space intensity distribution is given as follows:

11 $$ I\left({q}_x,{q}_z\right)={\left|{\displaystyle {\sum}_{\alpha, D}\frac{J_1\left(2\pi \frac{D}{2}\left({\tilde{q}}_x-{q}_x\right)\right)}{2\pi \frac{D}{2}\left({\tilde{q}}_x-{q}_x\right)}{\displaystyle \sum_t} \exp \left(2\pi i{q}_zc(t)\right)}\right|}^2{f}_1\left(\alpha \right){f}_2(D) $$

where, *J*_1_ is the first order Bessel function and *q*_*x*_ and *q*_*z*_ are the in-plane and out-of-plane components of the scattering vector; *α* and *D* are the out-of-plane orientation angle and lateral size of NW (or crystalline column in case of planar film); *f*_1_(*α*) and *f*_2_(*D*) are the Gaussian and log-normal distributions for *α* and *D*; *c*(*t*) is defined by Eq. (4); ${\tilde{q}}_x={q}_x\left(\alpha \right)$.

The Gaussian (Eq.(12)) and log-normal (Eq.(13)) distributions of *α* and *D* have to be used to correctly describe the broadening of the symmetrical and asymmetrical RSMs for III-nitride [14–17]:

12 $$ {f}_1\left(\alpha \right)=\frac{1}{\sigma_{\alpha}\sqrt{2\pi }} \exp \left(-\frac{\alpha^2}{2{\sigma_{\alpha}}^2}\right), $$

13 $$ {f}_2(D)=\frac{1}{D{\sigma}_D\sqrt{2\pi }} \exp \left(-\frac{{\left(lnD-\mu \right)}^2}{2{\sigma_D}^2}\right), $$

where, *σ*_*α*_ is the dispersion parameter in the *α* distribution; *μ* and *σ*_*D*_ are the location and scale parameters in the *D* distribution.

## Results and Discussion

It is well known that epitaxial films of III-nitrides grow in columnar structures of relatively perfect material bounded by dislocation arrays (the so-called mosaic model) [14–16]. Both the distribution of the out-of-plane orientation angles (*α*) and of the lateral sizes (*D*) of crystalline columns cause the broadening of symmetrical and asymmetrical RSMs. Since *α* and *D* broaden the RSMs normal to the diffraction vector and along the *q*_*x*_ axis, respectively, the two contributions can be separated only in an asymmetrical RSM [3]. Moreover, both the composition and the biaxial strain of graded Al_*x*_Ga_1 − *x*_N alloys affect the in- and out-of-plane lattice parameters and hence the peak position of asymmetrical RSMs [17]. Therefore, the fitting of an experimental asymmetrical RSM will allow for the separation of strain, composition, and structural parameters in graded Al_*x*_Ga_1 − *x*_N films and NWs.

**Fig. 4:**
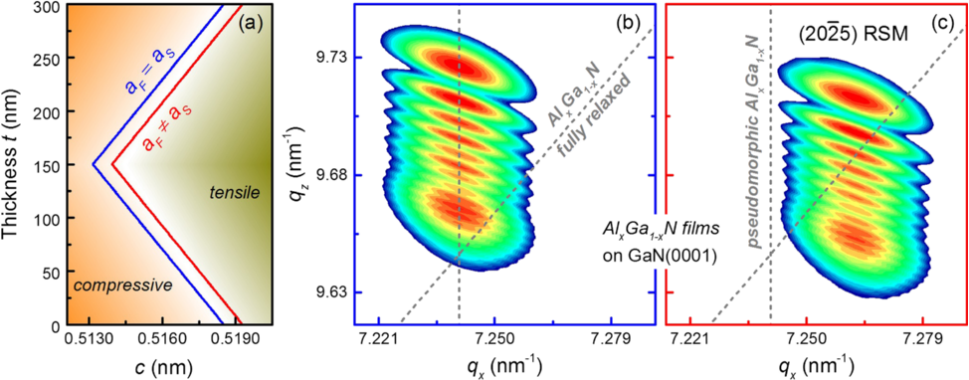
Calculated $\left(20\overline{2}5\right)$ RSMs of coherently strained, graded Al_*x*_Ga_1 − *x*_N films using the depth profiles of the out-of-plane lattice parameters shown in **a** where the *blue line* represents pseudomorphic growth and the *red* *line* partially relaxed growth. Pseudomorphic and partially relaxed growths on [0001]-oriented GaN substrates are simulated in **b** and **c**, respectively. The *gray dashed lines* indicate the position of pseudomorphic and fully relaxed growth

First, we apply the proposed model for graded Al_*x*_Ga_1 − *x*_N films by calculating the intensity distribution of $\left(20\overline{2}5\right)$ RSMs. For this, we use the *f*_1_(*α*) distribution for *a* ranges from −0.5 to 0.5 *deg* with *σ*_*α*_ = 0.05 in Eq. (12) and *f*_2_(*D*) distribution for *D* ranges from 30 to 300 nm with *σ*_*α*_ = 0.06 in Eq. (13). The *c*_*F*_(*t*) depth distributions of pseudomorphic (*a*_*S*_ = *a*_*F*_ = 0.31893 nm) and partially relaxed (*a*_*S*_ ≠ *a*_*F*_ = 0.318 nm) graded Al_*x*_Ga_1 − *x*_N films (Fig. 4a) were calculated according to Eq. (4) for the Al composition and in-plane strain profiles shown in Fig. 1. The calculation of $\left(20\overline{2}5\right)$ RSMs of graded Al_*x*_Ga_1 − *x*_N films was performed by Eq. (11) for the abovementioned structural and strain/compositional profiles and are shown in Fig. 4b, c.

As can be seen from Fig. 4b, c, coherent growth of both pseudomorphic and partially relaxed compositionally graded films results in continuous intensity distributions along the *q*_*z*_ axis of the $\left(20\overline{2}5\right)$ RSMs with fixed *q*_*x*_ positions indicating the relaxation degree of the film. This allows the in-plane lattice parameter of a whole graded Al_*x*_Ga_1 − *x*_N film to be determined from an experimental asymmetrical RSM; therefore, only the Al composition and strain profile have to be specified for the $\left(20\overline{2}5\right)$ RSM fitting.

**Fig. 5:**
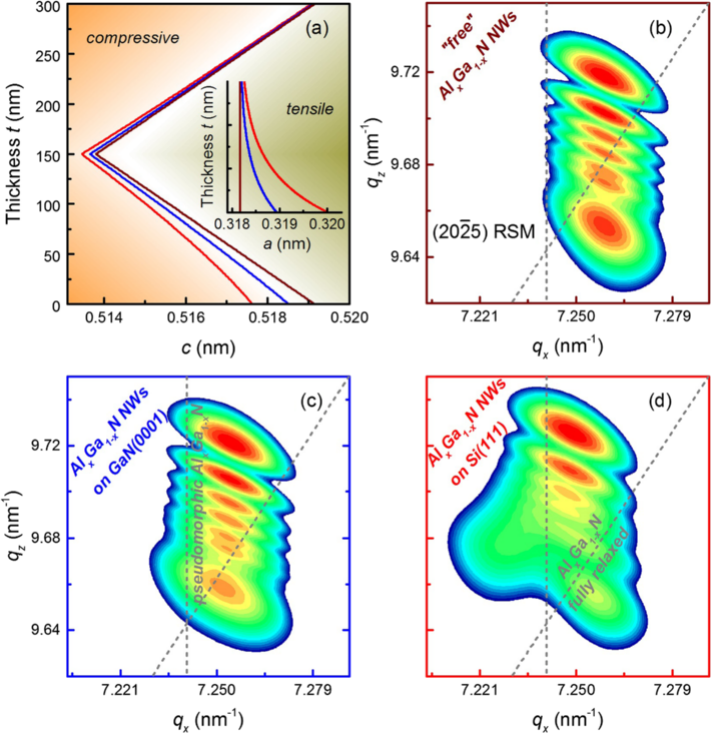
**a** The depth evolution of the out-of-plane lattice parameter, *c*_NW_(*t*), and the in-plane lattice parameter, *a*_NW_(*t*), (*inset*) of the graded Al_*x*_Ga_1 − *x*_N NWs grown “free standing” (*brown*), on GaN(0001) (*blue*), and on Si(111) (*red*). Calculated $\left(20\overline{2}5\right)$ RSMs of graded Al_*x*_Ga_1 − *x*_N NWs are shown in **b** for “free” growth, **c** for growth on GaN(0001), and in **d** for growth on Si(111). The *dashed gray lines* indicate the position of pseudomorphic (with respect to GaN) and fully relaxed Al_*x*_Ga_1 − *x*_N alloy for $\left(20\overline{2}5\right)$ reflection

Next, again using Eq. (11), we calculate the $\left(20\overline{2}5\right)$ RSMs of graded Al_*x*_Ga_1 − *x*_N NWs using the same *f*_1_(*α*) distribution as for graded Al_*x*_Ga_1 − *x*_N films, but now use a constant *f*_2_(*D*) = 100 nm, to represent the NWs. For this calculation we need the *a*_NW_(*t*) and *c*_NW_(*t*) depth profiles, which we calculate according to Eq. (10) and Eq. (4) for the Al composition and in-plane strain profiles shown in Fig. 1a and Fig. 3, respectively. The resulting lattice constant distributions are shown in Fig. 5a for the cases of graded Al_*x*_Ga_1 − *x*_N NWs on Si(111), GaN(0001), and for “free” NWs.

In Fig. 5, we see the evolution of the intensity distributions of $\left(20\overline{2}5\right)$ RSMs of graded Al_*x*_Ga_1 − *x*_N NWs as the substrate induced strain is reduced from growth on Si(111) to growth on GaN(0001) to growth of “free” NWs. As in the case of the coherently strained graded Al_*x*_Ga_1 − *x*_N films, the constant in-plane lattice parameter for “free” NWs (${\varepsilon}_{\left|\right|}^S(t)=0$ in Eq. (9)) gives rise to a typical intensity distribution along the *q*_*z*_ axis of the $\left(20\overline{2}5\right)$ RSM (Fig. 5b). In contrast, the exponential decay of the substrate induced strain, ${\varepsilon}_{\left|\right|}^S(t)$, from the NW’s bottom to the top (Fig. 3) results in the exponential in-plane lattice parameter relaxation (Fig. 5a, inset) and to a slope in the $\left(20\overline{2}5\right)$ RSM as shown in Fig. 5c and Fig. 5d for graded Al_*x*_Ga_1 − *x*_N NWs on GaN (with *a*_*s*_ = 0.31893 nm) and Si (*a*_*s*_ = 0.32 nm) substrates, respectively. Also, there are significant differences in RSMs for graded Al_*x*_Ga_1 − *x*_N NWs grown on GaN and Si substrates. As the difference in in-plane lattice constants from the base of the NW to the top becomes larger, we start to see a bifurcation of the lower lobes in the RSM as seen for the NW growth on the Si substrate in Fig. 5d. This is a unique feature of vertically aligned NWs, which exhibit large elastic relaxation along their length. This model is therefore not only very versatile in that it can be applied to NWs growing on different substrates, it is very powerful in that it can give definite lattice information along the entire length of the NW.

Finally, we want to pay attention to the maxima in the X-ray intensity distribution from both graded Al_*x*_Ga_1 − *x*_N films and NWs. These pronounced peaks, while very similar to in appearance, cannot be attributed to the Kiessig fringes alone, but rather to a coherent superposition of scattering vectors with appropriate lengths, as a result of the depth distribution of the concentration, *x*_Al_(*t*), in the graded Al_*x*_Ga_1 − *x*_N structure. Thereby, this shows the very high sensitivity of the RSMs to *x*_Al_(*t*) changes. Thus, the developed HRXRD method can be applied as a fast, non-destructive method for controlling the depth distribution of the Al with very high precision.

## Conclusions

In conclusion, we present a method for the efficient strain and composition depth profile determination of graded Al_*x*_Ga_1 − *x*_N films and NWs by fitting asymmetrical $\left(20\overline{2}5\right)$ RSMs. The peculiarities of strain relaxation are demonstrated for 300-nm thick coherent graded Al_*x*_Ga_1 − *x*_N films in comparison with partially relaxed graded Al_*x*_Ga_1 − *x*_N NWs. An internal strain due to Al composition variation and a substrate induced strain which exponentially relaxes from the NW’s bottom to the top were considered. We show that asymmetrical RSMs contain enough information for reliable strain and composition determination.
